# Higher antibody titres against *Pseudogymnoascus destructans* are associated with less white-nose syndrome skin lesions in Palearctic bats

**DOI:** 10.3389/fimmu.2023.1269526

**Published:** 2023-12-08

**Authors:** Jiri Pikula, Jiri Brichta, Veronika Seidlova, Vladimir Piacek, Jan Zukal

**Affiliations:** ^1^ Department of Ecology and Diseases of Zoo Animals, Game, Fish and Bees, University of Veterinary Sciences Brno, Brno, Czechia; ^2^ CEITEC: Central European Institute of Technology, University of Veterinary Sciences Brno, Brno, Czechia; ^3^ Institute of Vertebrate Biology, Czech Academy of Sciences, Brno, Czechia

**Keywords:** emerging wildlife infection, adaptive antifungal immunity, disease severity, indirect ELISA, antibody prevalence, *Myotis* bat species

## Abstract

**Introduction:**

Serological tests can be used to test whether an animal has been exposed to an infectious agent, and whether its immune system has recognized and produced antibodies against it. Paired samples taken several weeks apart then document an ongoing infection and/or seroconversion.

**Methods:**

In the absence of a commercial kit, we developed an indirect enzyme-linked immunosorbent assay (ELISA) to detect the fungus-specific antibodies for *Pseudogymnoascus destructans*, the agent of white-nose syndrome in bats.

**Results and Discussion:**

Samples collected from European *Myotis myotis* (n=35) and Asian *Myotis dasycneme* (n=11) in their hibernacula at the end of the hibernation period displayed 100% seroprevalence of antibodies against *P. destructans*, demonstrating a high rate of exposure. Our results showed that the higher the titre of antibodies against *P. destructans*, the lower the infection intensity, suggesting that a degree of protection is provided by this arm of adaptive immunity in Palearctic bats. Moreover, *P. destructans* infection appears to be a seasonally self-limiting disease of Palearctic bats showing seroconversion as the WNS skin lesions heal in the early post-hibernation period.

## 1 Introduction

Novel and emerging wildlife infections that threaten biodiversity, domestic animals and/or humans are of great interest to researchers seeking to gain insights into host-pathogen interactions (1). In such cases, risks of infection are driven by multiple factors, including host-pathogen co-evolution and dynamics, life history traits, community structure of reservoir hosts, transmission rate and dispersal of the agent and environmental change (2, 3). Understanding the immune responses of bats, which are recognized as reservoir hosts of zoonotic agents, has recently become a critical issue to identify mechanisms that allow pathogen circulation and emergence of severe infections (4). However, the majority of articles on this subject concentrate on viruses of bats and mention the extraordinary ability of chiropterans to cope with RNA viral infections (5–11); consequently, much less is known about bat immunity against non-viral pathogens and/or pathogens that cause clinically manifesting diseases in bats (6).

The need for addressing gaps in our knowledge of bat immunity has also been highlighted by conservation concerns associated with the emergence of white-nose syndrome (WNS), a major threat to naïve bat species in North America (12–14). The causative fungal agent of WNS, *Pseudogymnoascus destructans*, has proven to be devastating for hibernating insectivorous bats in the Nearctic; however, it appears to be tolerated by Palearctic bats (15), with only sporadic cases of WNS-associated fatality being documented in Europe (16, 17), despite hyperendemic (i.e., highly prevalent and persistent) exposure to this psychrophilic skin invasive pathogen in contaminated hibernacula. The high prevalence and infection intensity (measured as fungal load and number of skin lesions) of WNS without mass mortality suggests an equilibrium in host-pathogen interactions between Palearctic bats and *P. destructans* (15). Exceptional infection tolerance in bats is thought to be due to a balance between protective and pathologic immune responses mediated through pro- and anti-inflammatory cytokines (4, 6, 18, 19).

Exposure of a bat to *P. destructans* may result in an invasive skin infection, with extensive damage to its flight membranes (16, 20, 21) and severe disruption of the effective skin barrier function explaining the pathophysiology of the disease (22–25), resulting in altered torpor patterns, increased arousal frequency, premature depletion of fat reserves and dehydration during hibernation (26). Enzymes secreted by *P. destructans*, e.g., destructin-1 peptidase, subtilisin-like serine peptidase and lipases, enable the fungus to invade and digest cutaneous tissues (27, 28). On histopathology of the flight membrane, specifically distinctive lesions include both cupping erosions and/or full-thickness invasion, where the fungus breaches the skin basement membrane (16, 20, 21), the WNS skin lesions being loaded with hyperaccumulated riboflavin, a secondary fungal metabolite emitting an intensive orange-yellow fluorescence following excitation with 366-385 nm ultraviolet (UV) light (29). This fluorescence can be used as a non-lethal diagnostic method to identify bats showing WNS skin lesions in their UV-trans-illuminated wing membranes (30).

Analysis of blood parameters in greater mouse-eared bats (*Myotis myotis*) has revealed a threshold of ca. 300 skin lesions on both wings induced by *P. destructans*, combined with suboptimal hibernation conditions, that distinguish healthy hibernating bats from those with disruption of body homeostasis (31). Pathophysiological effects of WNS in European bats tend to manifest as a mild metabolic acidosis, decreased blood glucose and peripheral blood eosinophilia. Bats displaying blood homeostasis disruption had a lower body mass index (BMI) and hibernated with a 2°C lower body surface temperature (31).

Note, however, there is still some discrepancy about the presence and/or absence of a systemic response to WNS infection in European bats. While it has been detected at both the organismal (31) and transcriptional (32) levels, it has not been recognized in the plasma proteomic profile (33). Likewise, a study of adaptive immunity in two European bat species detected no antibodies against *P. destructans* in winter, and only low titres in spring, concluding that antibody-mediated immunity cannot explain the survival of European bats infected with the WNS fungus (34). These differences in findings on European bat responses may or may not be due to differences in WNS status and the severity of infection in individuals selected for the studies.

In response to these conflicting findings, the objective of the present study was to develop an enzyme-linked immunosorbent assay (ELISA) for measuring antibody response to *P. destructans* infection based on antigens produced by pathogenic fungal strain isolates from North America, Europe and Asia. We predicted that Palearctic bats would show differences in antibody prevalence and titres against *P. destructans* in relation to species, their age and infection severity at the time of examination. We then tested surviving bats in the early post-hibernation period to assess whether there was a rise in antifungal antibody titre of WNS infection.

## 2 Materials and methods

### 2.1 Ethics statement

Bats were sampled in the field in accordance with Czech Law No. 114/1992 on Nature and Landscape Protection, based on permits 1662/MK/2012S/00775/MK/2012, 866/JS/2012 and 00356/KK/2008/AOPK issued by the Czech Agency for Nature Conservation and Landscape Protection. Sampling in caves in the Ural Mountains (Russia) was approved by the Institute of Plant and Animal Ecology, Ural Division of the Russian Academy of Sciences (No. 16353–2115/325), and the Tyumen State University (No. 06/162). Experimental procedures were approved by the Ethical Committee of the Czech Academy of Sciences (No. 169/2011). All authors were authorized to handle free-living bats under Czech Certificate of Competency No. CZ01341 (§17, Act No. 246/1992).

### 2.2 Sample collection and bat examination

We sampled a total of 46 bats, comprising 35 greater mouse-eared bats (*Myotis myotis*) from the Czech Republic and 11 pond bats (*Myotis dasycneme*) from Russia. Sampling was performed at two bat hibernation sites in the Czech Republic (the Šimon and Juda mines in the Jeseníky Mountains and the Mořina Quarry in Bohemia) in 2018 (April) and at three hibernacula in the Russian Ural Mountains (Arakaevskaja, Komsomolskaja and Partizanskaja caves) in 2017 (April). The sex of each bat was determined and the age estimated based on epiphyseal ossification of the thoracic limb fingers and tooth abrasion (35). We also measured the forearm length using callipers and body mass using a portable top-loading balance. BMI was determined as a bat’s body mass (g) divided by the left forearm length in mm (36). To examine the status and intensity of *P. destructans* infection, we took a swab of each bat’s wing surface (FLOQ Swabs, Copan Flock Technologies s.r.l, Italy) for later examination in the laboratory, where presence of the fungus was tested using polymerase chain reaction (PCR) and WNS skin lesions were manually enumerated from photographs of both wings taken over a 368 nm ultra-violet (UV) lamp using the individual object counting tool of ImageJ, as described elsewhere (30, 31). A wing membrane biopsy targeting fluorescing lesions with a 4 mm sterile punch (Kruuse, Denmark) was collected from each bat under UV guidance to check for invasive fungal growth distinctive for WNS on histopathology (16).

To measure titres of antibodies against *P. destructans*, samples of blood were collected from bats ca. 60 minutes after capture, this providing the re-warming period necessary for efficient blood flow from punctured veins under field conditions of late hibernation. The procedure of blood collection comprised skin surface disinfection with an alcohol pad, the spreading of a small drop of heparin over the intended skin puncture site and puncturing of the uropatagial vessel with a sterile needle to obtain a 100μl blood sample using a heparinised pipette tip. The puncture site was then sealed using a drop of surgical absorbable tissue glue (Surgibond, SMI AG, Belgium) to stop further bleeding. All bats were handled gently and, prior to release at the hibernaculum, provided orally with 5% glucose and physiological saline solution for rapid replenishment of energy and fluids. The whole blood samples were centrifuged 15 min at 1500g to separate the plasma, which was then stored at -80°C until further use in antibody measurements.

Eleven adult *M. myotis* bats (three female, eight male) from the Šimon and Juda hibernation site were kept in captivity for four weeks to obtain paired blood samples in order to test for a rise in the antifungal antibody titre of WNS infection surviving bats in the natural early post-hibernation period (i.e., in April to May). These bats were housed at a temperature of 21°C in an indoor flight chamber with soft mesh on the inner walls, cloth layers to provide roosting and hiding places and humidifiers to maintain humidity between 60 and 70%. The bats were exposed to a natural day and night cycle, provided with a water dish for drinking and encouraged and taught to self-feed on mealworms and crickets.

### 2.3 ELISA for measuring antibodies against *Pseudogymnoascus destructans*

Six pathogenic strain isolates of *P. destructans* from little brown bats (*Myotis lucifugus*), *M. myotis*, common long-eared bats (*Plecotus auritus*) and *M. dasycneme* infected in North America, Europe and Asia, listed in (29) as 20631-21^T^, CCF3941, CCF3943, CCF4103, CCF4987 and CCF4986 with ITS rDNA accession numbers EU884921, HM584956, HM584957, LN852366, LN852358 and LN852359, respectively, were used to develop an ELISA for detection of antifungal antibodies in cooperation with the Biovendor Research and Diagnostic Product Department (Brno, Czech Republic). The fungal strains were cultivated on sterile Sabouraud dextrose agar plates wrapped in Parafilm (Fisher Scientific, USA) after inoculation at 10°C in darkness. After eight weeks of colony growth, phosphate buffered saline (PBS; Sigma-Aldrich, Germany) was used to harvest the fungal conidia. Next, the number of *P. destructans* fungal elements was determined in a Bürker counting chamber (Fisher Scientific, USA), inactivated with formaldehyde (Roth, Germany), sonicated in a Sonorex Super RK 156 BH sonic bath (Bandelin, Germany) and then used to coat 96-well microtitration plates (Nunc Inc., Denmark) in 0.1M carbonate buffer (pH 9.6; Merck Sigma-Aldrich, Germany). An equal coating ratio of all six *P. destructans* pathogenic strains was used in each well. The carbonate buffer used for microtitration plate coating contained 50 000 conidia per 1mL. Each well was treated with a volume of 100µL of the coating solution. After 48h of coating and immobilization at 4–6°C, the plates were washed and blocked to suppress non-specific binding and stabilized in sucrose solution (Sigma-Aldrich) for 1h before drying, sealing under vacuum in the presence of silica desiccant (Roth, Germany) and storing at 2–8°C until use.

Prior to antibody measurement, bat sera were PBS diluted 1:100, then placed into the testing wells and incubated in the plates for 1h at 25°C, after which a two-step protocol was used for detection of anti-*P. destructans* specific antibodies. The first step included addition of biotinylated monoclonal anti-bat antibody (Goat anti-Bat IgG Heavy and Light Chain Antibody Biotinylated, Bethyl Laboratories, USA) and incubation for 1h. This was followed by addition of streptavidine-poly HRP (horseradish peroxidase) conjugate (Mir Biotech s.r.o., Czech Republic) and 1h of incubation. In each case, a Multi Bio 3D Mini Shaker (Biosan, Latvia) set at 300rpm was used during the period of incubation. The microtitration plates were washed four times with PBS containing 0.05% of Tween-20 (Sigma-Aldrich) in-between the incubation steps. A color reaction was developed through addition of a reagent peroxide and TMB substrate (3,3′,5,5′-tetramethylbenzidine; Sigma-Aldrich) and stopped with 1M sulphuric acid (Sigma-Aldrich). IgG (0.01% solution) in Tris bovine serum albumin (BSA) buffer (Sigma-Aldrich) was used as a blank, and plasma samples collected from 12 noctule bats (*Nyctalus noctula*) in a non-related study (37) served as a negative control, this species being a non-cave hibernator that is not exposed to the *P. destructans* fungus. The intensity of *P. destructans* specific antibody binding was detected through duplicate dual wavelength measurements on an Elx808 ELISA microplate Reader (BioTek, USA), with 630nm absorbance serving for subtraction of optical non-homogeneity and 450nm for measuring the specific signal, the absorbance values being measured in duplicates and multiplied by the plasma dilution factor (x100) to obtain antifungal antibody titres. Paired blood samples of *M. myotis* bats, positive for WNS skin lesions and showing a rise in the antifungal antibody titre after healing from the WNS infection in the early post-hibernation period, were considered as positive controls.

### 2.4 Statistical analysis

All statistical analysis was undertaken using the TIBCO Statistica^®^ software package v.14.0.0 (TIBCO Software Inc., USA). Normal distribution of variables was tested using the Shapiro-Wilk test. As WNS UV lesion counts and antibody titres were not normally distributed, these variables were log transformed (log n+0.5 and log n, respectively), re-checked for normality and used for all statistical analyses. Differences between means of variables (WNS UV lesion count, antibody titre and relative increase in antibody titre) were tested using the t-test for independent and/or paired samples. As there were no differences in BMI values between female and male and adult and subadult *M. myotis* (t = -0.154, p = 0.879 and t = 0.011, p = 0.991, respectively), the data were pooled for subsequent analyses. The effect of WNS UV lesion count on antibody titre was tested using univariate general linear model (simple regression) separately for each bat species. The Pearson correlation was used as a measure of linear correlation of variables.

## 3 Results

PCR examination confirmed *P. destructans* infection in 100% of *M. myotis* and *M. dasycneme* bats and histopathological findings matched WNS diagnostic criteria in all *M. myotis* bats examined. WNS prevalence in *M. dasycneme* bats was 81.82% based on UV skin lesion detection and histopathology (**Table 1**). Severity of infection ranged between 99 and 2949 skin lesions in Czech *M. myotis* bats and from 0 to 625 skin lesions in Russian *M. dasycneme* bats. All bats of both species were seropositive for anti-*P. destructans* antibodies (**Table 1**).

**Table 1:** Characteristics of bats examined for antibodies against *Pseudogymnoascus destructans*.

Bat species	*Myotis myotis*				*Myotis dasycneme*
**Age**	Adult n=27		Subadult n=8		Adult n=11
**Gender**	Female n=5	Male n=22	Female n=1	Male n=7	Female n=11
**Body mass [g]**	19 – 28	20.0 – 26.5	18.5	19 – 27	13.5 – 19
**Antebrachium length [mm]**	60.0 – 64.4	56.7 – 64.4	61.6	56.3 – 65.2	45.9 – 48.5
**Body mass index**	0.31 – 0.47	0.33 – 0.44	0.3	0.32 – 0.44	0.28 – 0.41
**WNS UV lesions**	139 – 454	99 – 2949	2318	143 – 2462	0 – 625
**Antibody titre I**	0.113 – 0.658	0.047 – 1.223	0.151	0.151 – 0.551	0.086 – 0.648
**Antibody titre II**	0.370 – 1.904	0.151 – 0.58	0.316	0.191 – 0.541	N.A.

*Myotis myotis* and *Myotis dasycneme* bats were sampled in the Czech Republic and Russia in 2017 and 2018. Quantitative characteristics are given as minimum and maximum values. WNS UV lesions, white-nose syndrome skin lesions in both wing membranes, identified and enumerated using trans-illumination with 366-385 nm ultraviolet light; Antibody titre I, values based on blood samples collected in the field towards the end of the hibernation period (April); Antibody titre II, values based on blood samples of WNS infection-surviving bats (n=11) after four weeks of captivity in the early post-hibernation period; N.A., not available.

**Figure 1:**
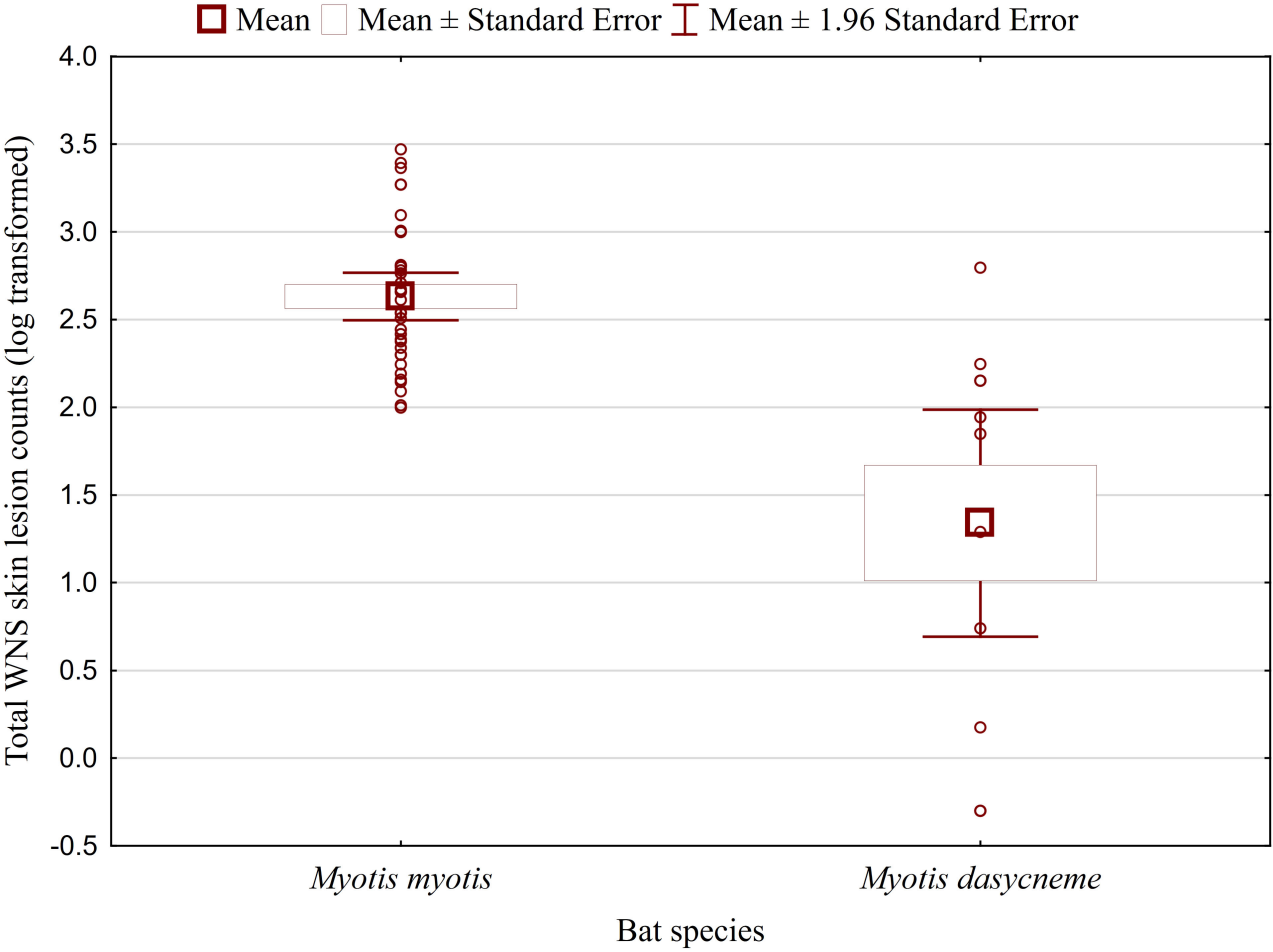
WNS UV lesion counts on both wings of *Myotis myotis* and *Myotis dasycneme* bats sampled in the Czech Republic and Russia in 2017 and 2018. WNS UV lesions = white-nose syndrome skin lesions identified and enumerated using trans-illumination with 366-385 nm ultraviolet light. *Myotis myotis* and *Myotis dasycneme* bats differed significantly in WNS UV lesion counts (t = 5.897, p < 0.001).

**Figure 2:**
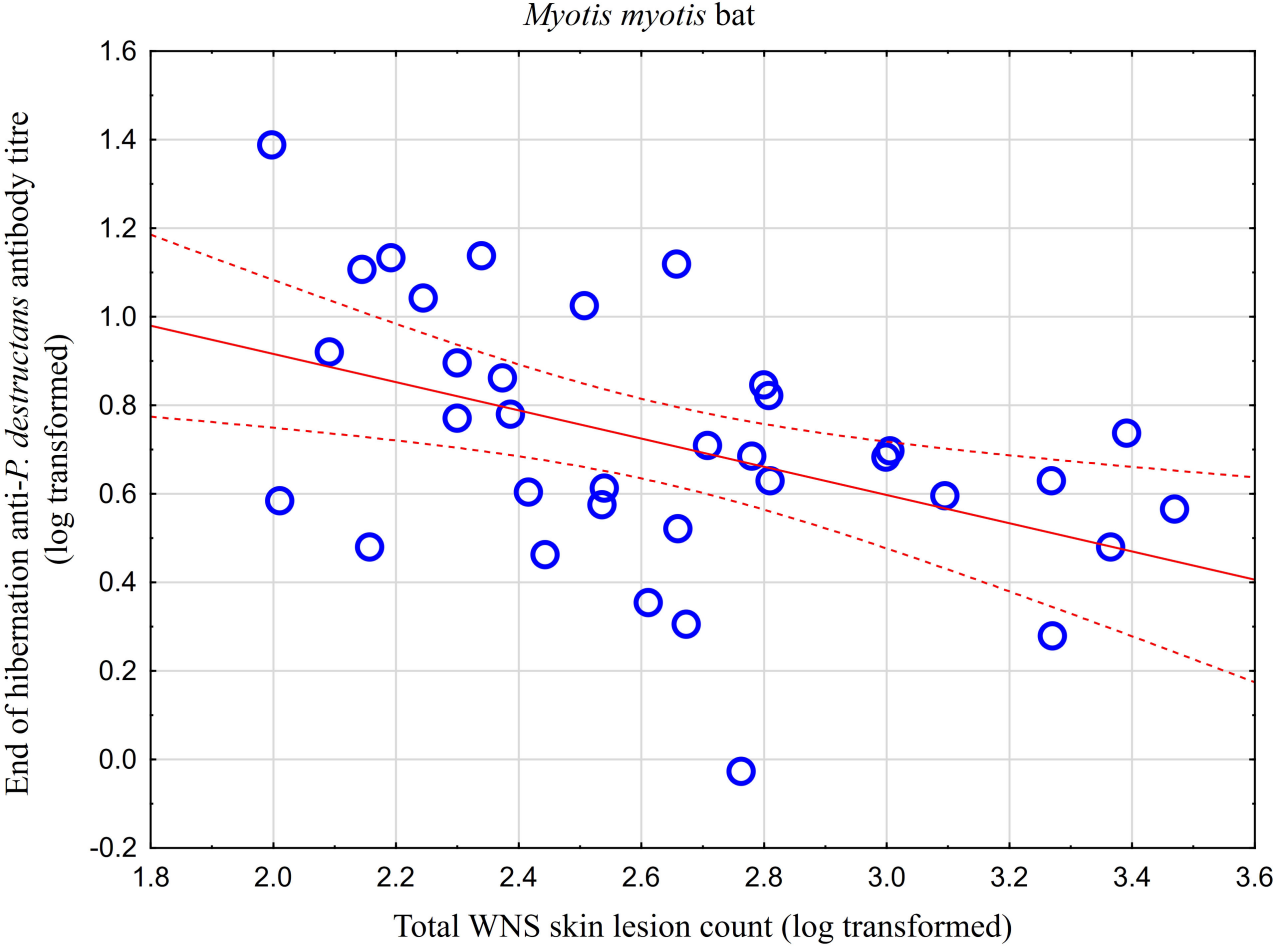
Relationship between WNS UV lesion counts and antibody titres measured in blood samples collected towards the end of the hibernation period (April). Linear regression with 95% confidence intervals. A significant relationship was only confirmed for *M. myotis* (F = 8.512, p = 0.006) with a significant negative correlation (r = -0.453, p = 0.006). Higher antibody titres against *Pseudogymnoascus destructans* were associated with less white-nose syndrome skin lesions.

**Figure 3:**
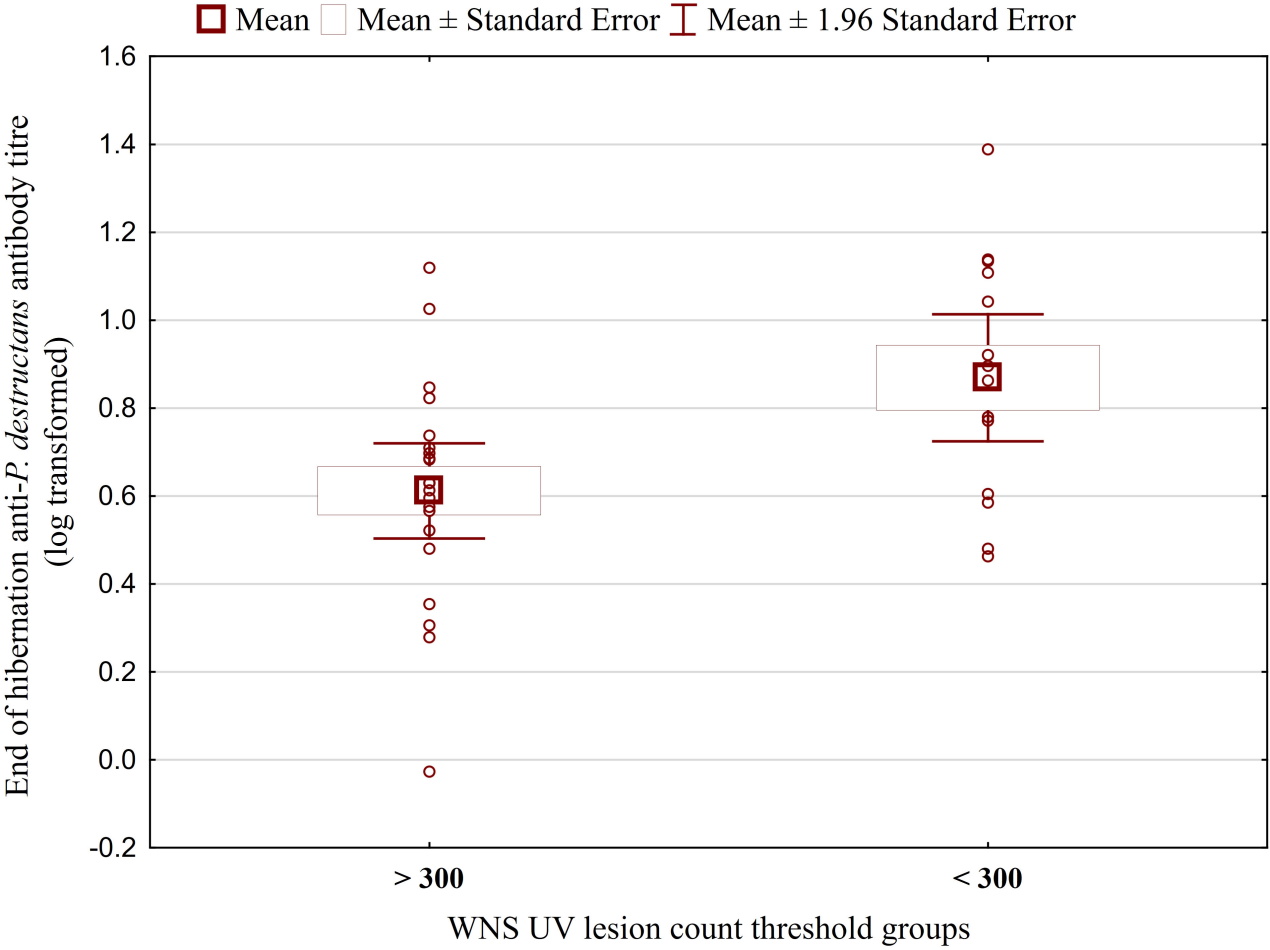
Antibody titres against *Pseudogymnoascus destructans* in *Myotis myotis* bats grouped based on a threshold of 300 WNS skin lesions on both wings. After dividing the *M. myotis* data into two groups based on the threshold of 300 skin lesions on both wings (the point at which WNS affects the bat’s health status), significantly lower antibody titres (t = -2.847, p = 0.008) were observed in the > 300 lesion group. For bats > 300 n=21, for bats < 300 n=14.

While no significant difference was observed between bat species in their antibody titres (t = 0.413, p = 0.681), they differed significantly in WNS UV lesion counts (t = 5.897, p < 0.001; **Figure 1**). There were no differences in antibody titres even when just the subset of females was compared (t = 0.925, p = 0.370 and t = 2.705, p = 0.016, respectively), with female *M. dasycneme* having significantly fewer skin lesions (mean = 115) than *M. myotis* (mean = 676). A significant relationship between WNS UV lesion count and antibody titre was only confirmed for *M. myotis* (F = 8.512, p = 0.006) with a significant negative correlation (r = -0.453, p = 0.006; **Figure 2**). After dividing the *M. myotis* data into two groups based on the threshold of 300 skin lesions on both wings (the point at which WNS affects the bat’s health status), significantly lower antibody titres (t = -2.847, p = 0.008) were observed in the > 300 lesion group (**Figure 3**).

**Figure 4:**
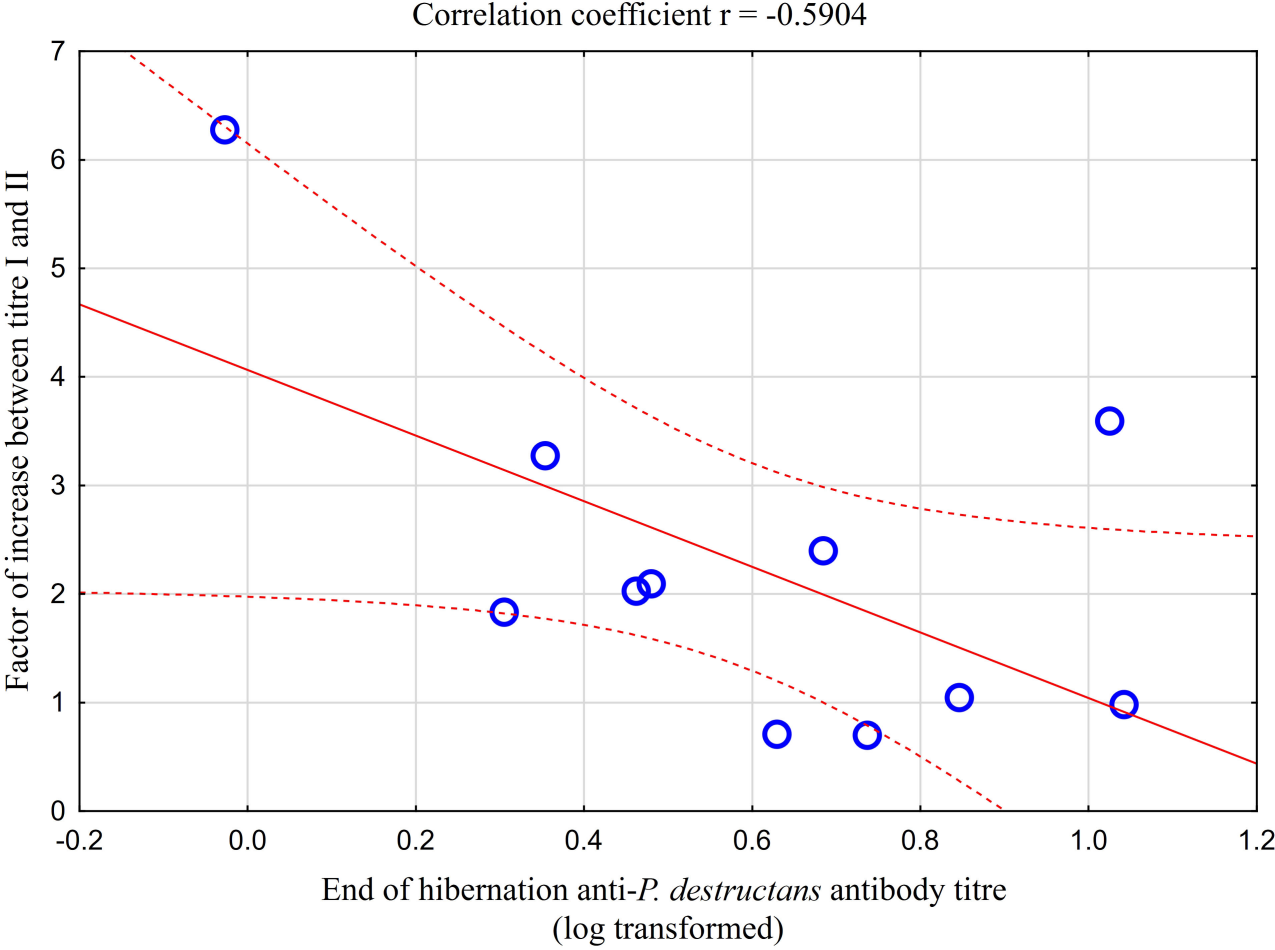
Relationship between relative increase in antibody titres in captive bats after four weeks of euthermy (titre II) and antifungal antibodies measured in the same individuals at the end of the hibernation period (titre I). Linear regression with 95% confidence intervals. The relative increase in antibody titres was negatively, though non-significantly, correlated with titre values obtained during the first measurement (r = -0.590, p = 0.056).

Comparison of paired titres for antifungal antibodies measured in the same *M. myotis* individuals at the end of the hibernation period and after four weeks of euthermy in captivity revealed a significant positive rise in all individuals, except for three bats showing a minor titre decrease of up to 30%. A total of 64% of bats showed a 2- to 6-fold seroconversion, with the relative increase in antibody titres being negatively, though non-significantly, correlated with titre values obtained during the first measurement (r = -0.590, p = 0.056; **Figure 4**). Adult bats exhibited an average 2.87-fold seroconversion of antibody titres during the four-week period. While there was no difference in the relative increase between adult females and males, subadult females displayed a significantly higher relative increase in antibody titres than subadult males (t = -5.568, p = 0.031).

## 4 Discussion

### 4.1 *P. destructans* infection status at the study sites

Our study combined detection of *P. destructans* infection in Palearctic bats using standard procedures for non-lethal examination, such as PCR (38), wing membrane trans-illumination with a modified Wood’s lamp (30) and histopathology of skin lesions collected under UV guidance (16, 20) with a newly developed ELISA for measuring antibodies against *P. destructans*. Data gathered in the present study correspond with earlier reports on bat species-specific prevalence, with levels ranging from 64 to 100% (15, 39). The knowledge gained on infection status and severity in this study can be seen as a major advance. On the other hand, it is our opinion that the unknown *P. destructans* infection status of bats collected from Germany and Finland by Johnson et al. (34), along with their relatively small sample size, may limit the validity of their results and conclusions drawn as regards European bats.

### 4.2 Bats produce antibodies against *P. destructans*

Contrary to the findings of Johnson et al. (34), the results of the present study indicate that European *M. myotis* bats respond to *P. destructans* infection by producing antibodies. Antibody findings in both European *M. myotis* bats and Russian *M. dasycneme* bats may be similar to naturally infected *M. lucifugus* bats in North America showing the highest titres in regions with longer histories of WNS (34). Even subadult *M. myotis* individuals tested seropositive, indicating that they responded to the fungal infection during their first hibernation winter. Lorch et al. (40) showed experimentally that clinical signs and skin infection with lesions diagnostic of WNS develop within 83 to 102 days of hibernation and the disease peaks at the end of hibernation period (41). The time-course of antibody response to *P. destructans* infection in bats is rather unknown. However, for example in human histoplasmosis, antifungal antibodies generally take up to eight weeks to reach detectable levels (42). As the bat winter hibernation period lasts from five to six months (43, 44), this provides ample time for disease progression and an immune system response. Clearly, detection of seropositive bats does not mean that antibodies will be present indefinitely; however, it is still unclear whether the antibody titres against *P. destructans* remain to the next hibernation winter, and what antibody levels are associated with the threshold of protection. Normally, one might assume that antibodies in Palearctic bats would decline in the absence of further fungal challenge in summer and, indeed, Johnson et al. (34) documented higher anti-*P. destructans* titres in spring-sampled North American bats than in summer. On the other hand, the high prevalence of antibodies in Palearctic bat populations may reflect either a high rate of infection or a low rate of antibody loss, with repeated challenges every winter. Likewise, there have been no studies on antibody titres in lactating females and potential antibody transfer in the colostrum, and little is known about factors that stimulate seroconversion in some individuals and seroreversion in others. One may hypothesize that both infection intensity and severity (16) and bat health status (31) are correlates of the immune response. Interestingly, two *M. dasycneme* bats in this study displayed antibodies despite being negative on UV skin lesion detection and histopathology, though both bats subsequently tested positive for the *P. destructans* fungus on PCR examination, suggesting that their antibodies developed rather in response to infection during the previous winter hibernation.

### 4.3 Fungal immunogenicity

The design of the ELISA kit used in this study was based on fungal conidia triggering stronger immunogenic responses than mycelial components (45). The immune system of *P. destructans*-infected bats is, however, mainly exposed to fungal hyphae invading deeper skin layers and secondary metabolites produced by the fungus. Conidia predominate on the skin surface because they are produced by aerial hyphae that colonize skin surface, meaning that the immune system is less exposed to conidia (16). As little is known about differences in the immunogenicity of pathogenic *P. destructans* strains isolated from bats over extensive geographic areas of the Palearctic and Nearctic regions, our ELISA kit was produced using antigens of six pathogenic strains to cover the available genetic variability of the fungus, including its mating types (15, 29). This is a similar strategy to that used in the study of Brennan et al. (46), who also tested multiple *P. destructans* strains to determine ELISA specificity. Brennan et al. (46), using immunofluorescence antibody testing, also demonstrated that various *Pseudogymnoascus* species share a conserved immunoreactive antigen, while Carvalho et al. (47) showed that the immunogenic stimuli of fungi depend on the ability of the innate immune system to sense pattern recognition receptors. Interestingly, this suggests that the immunogenic antigens of *P. destructans* that stimulate antifungal immunity, i.e., calnexin and destructin-1, could be used to prepare a vaccine promoting protection against WNS (48).

### 4.4 Immune suppression and immune response to fungal infection in bats

Seasonality is a driver of the annual cycle of temperate insectivorous bat species (49), and governs the year-round dynamics of most physiological functions, including the immune response (50). In winter, hibernating bats are subject to physiological extremes as they go through cycles of prolonged torpor with shorter periods of arousal (51–53). During winter hibernation torpor, bats lower their body temperature close to the ambient temperature, which tends to range between 0 and 12°C in hibernacula of temperate vespertilionids and rhinolophids (54, 55). As body temperature is an important factor modulating bat immune defense mechanisms (34, 37, 56–61), they may face reduced innate and adaptive functions in winter (57–59, 62). While immune suppression enhances fungal pathogenesis, fungal infections such as WNS will threaten such immune-compromised hosts (47, 63, 64).

Our results indicate that immune suppression of Palearctic bats during winter hibernation allows invasive infection of living skin tissues by the fungal agent, manifesting as distinctive WNS lesions. At the same time, the degree of immune suppression does not prevent production of antibodies against the invading agent. The immune system becomes more effective in the production of antifungal antibodies in the post-hibernation period, as documented by the comparison of paired titres measured in the same *M. myotis* bat individuals at the end of the hibernation period and after four weeks of natural euthermy.

The filamentous growth of fungal pathogens represents a particular challenge for the immune system (65). Neutrophils represent the first line of defense against fungi and are capable of modifying the overall immune response (66). WNS cupping erosions on bat wings, for example, heal through marked neutrophilic inflammation and sequestration of the fungal agent from the skin (16). Hibernating North American *M. lucifugus* bats infected with *P. destructans* express genes associated with local inflammation in the skin (67) and bat populations sampled prior to and after exposure to the WNS agent show a shift in immunogenetic diversity (68). During hibernation, bats may balance their inflammatory response to fungal exposure through energy-saving strategies (69). Regulation of gene expression has also been noted in the fungal agent as a response to the host-pathogen interaction during the course of WNS infection (70). Importantly, macrophages derived from *M. myotis* have been shown *in vitro* to be able to increase functional performance within minutes of transition from torpor temperatures to euthermic arousal (61).

In practice, however, a combination of cell-mediated and humoral responses is often necessary for clearance of fungal infections (47, 63, 65, 71). Antibodies may then participate in protection against fungal diseases through direct action on fungal cells and cytotoxicity, enhancement of phagocytosis and/or complement activation (65). Antibody-mediated immunity against fungi may even be deleterious (65). In North American *M. lucifugus* bats, for example, a cell-mediated delayed-type hypersensitivity was shown to kill WNS fungus-infected bats weeks after emergence from hibernation (56). Paradoxically, therefore, antifungal immunity can range over a continuum from deficiency to hyperactivity (47). In the present study, WNS infection surviving adult *M. myotis* bats held in captivity in the early post-hibernation period showed no signs of immune-mediated pathology associated with post-emergent healing of wing membrane lesions.

### 4.5 Can measurements of bat antibody titres against *P. destructans* be used in further studies?

While the direct conservation risks of emerging wildlife infections are clear, the adverse carry-over effects that reduce the success of such conservation efforts are often complex and difficult to document (72). Theory defines carry-over effects in wild animals as non-lethal biological processes that act in one season and influence performance in the next season (73), with carry-over phenomena believed to occur at the individual and/or population or community levels. Studies dealing with carry-over effects measure different indicators of condition and/or health (e.g., body mass, fat mass, pathogen load, blood profile, metabolic rate, immune defense) at the end of one season as a proxy for explaining subsequent variations in fitness, including reproduction and survival.

Very little is known about the health of European *P. destructans*-infected hosts in the period following emergence from hibernation. Upon arousal, early euthermic females may face a trade-off between mounting an immune response and the energetic investment needed to initiate gestation (74, 75). North American bat species recovering from *P. destructans* infection have shown shifts in pregnancy and lactation, suggestive of reproductive fitness consequences (76).

The characteristics of WNS make it an ideal model for examining carry-over effects associated with pathogen pressure (77), as 1) the infection is seasonally limited, 2) all bats roosting in a contaminated hibernaculum are exposed to the pathogen, 3) WNS survivors recover in the post-hibernation period and clear the infection, and 4) WNS pathogen pressure can be quantified, either as fungal load (infection intensity), through image analysis of wing membrane damage and/or through histopathology grading (disease severity) based on non-lethal skin biopsies (16).

Of all the European bat species, *M. myotis* shows the highest prevalence and infection intensity with WNS fungus (15, 16, 21, 31), making it the most suitable model species for studying immune responses and carry-over effects. In the same context, *M. dasycneme* may be used for host-pathogen studies encompassing Palearctic regions outside *M. myotis* distribution (15, 78). An important issue, however, is how to link pathogen pressure during bat hibernation with indicators of condition and health in the post-hibernation period. We argue that comparisons of antibody titres against *P. destructans* infection are a suitable candidate for obtaining such a WNS disease-specific link.

## 5 Conclusions

The WNS fungus, *P. destructans*, is endemic throughout temperate regions of Europe and Asia, meaning that hibernating insectivorous bats are naturally exposed to this skin-infecting pathogen every winter. Interestingly, reports on histopathology indicate equal findings of focal skin-tissue invasiveness distinctive for WNS lesions in Palearctic and Nearctic bats (15, 16). Here, we show highly prevalent antibodies against *P. destructans* in Palearctic bats, a response pattern indicating an inverse relationship between antibody titre and skin lesion count and an increase in antibody titres in the early post-hibernation period. Further studies will be needed to obtain deeper insights into how both the innate and adaptive arms of immunity contribute to the survival of Palearctic bats exposed to the virulent WNS fungus.

## Data availability statement

The raw data supporting the conclusions of this article will be made available by the authors, without undue reservation.

## Ethics statement

The animal study was approved by Ethical Committee of the Czech Academy of Sciences. The study was conducted in accordance with the local legislation and institutional requirements.

## Author contributions

JP: Conceptualization, Data curation, Formal analysis, Funding acquisition, Investigation, Methodology, Supervision, Writing – original draft, Writing – review & editing. JB: Conceptualization, Methodology, Validation, Writing – review & editing. VS: Data curation, Investigation, Methodology, Validation, Writing – review & editing. VP: Investigation, Methodology, Writing – review & editing. JZ: Conceptualization, Data curation, Formal analysis, Funding acquisition, Investigation, Methodology, Supervision, Writing – review & editing.

## References

[1] Daszak P Cunningham AA Hyatt AD. Emerging infectious diseases of wildlife– threats to biodiversity and human health. Science (2000) 287:443–9. doi: 10.1126/science.287.5452.443 10642539

[2] Daszak P Cunningham AA Hyatt AD. Anthropogenic environmental change and the emergence of infectious diseases in wildlife. Acta Trop (2001) 78:103–16. doi: 10.1016/S0001-706X(00)00179-0 11230820

[3] Keesing F Belden LK Daszak P Dobson A Harvell CD Holt RD. Impacts of biodiversity on the emergence and transmission of infectious diseases. Nature (2010) 468:647–52. doi: 10.1038/nature09575 PMC7094913 21124449

[4] Mandl JN Ahmed R Barreiro LB Daszak P Epstein JH Virgin HW. Reservoir host immune responses to emerging zoonotic viruses. Cell (2015) 160:20–35. doi: 10.1016/j.cell.2014.12.003 25533784 PMC4390999

[5] Brook CE Dobson AP. Bats as ‘special’ reservoirs for emerging zoonotic pathogens. Trends Microbiol (2015) 23:172–80. doi: 10.1016/j.tim.2014.12.004 PMC7126622 25572882

[6] Mandl JN Schneider C Schneider DS Baker ML. Going to bat(s) for studies of disease tolerance. Front Immunol (2018) 9:2112. doi: 10.3389/fimmu.2018.02112 30294323 PMC6158362

[7] Banerjee A Baker ML Kulcsar K Misra V Plowright R Mossman K. Novel insights into immune systems of bats. Front Immunol (2020) 11:26. doi: 10.3389/fimmu.2020.00026 32117225 PMC7025585

[8] Calisher CH Childs JE Field HE Holmes KV Schountz T. Bats: important reservoir hosts of emerging viruses. Clin Microbiol Rev (2006) 19:531–45. doi: 10.1128/CMR.00017-06 PMC1539106 16847084

[9] Schountz T Baker ML Butler J Munster V. Immunological control of viral infections in bats and the emergence of viruses highly pathogenic to humans. Front Immunol (2017) 8:1098. doi: 10.3389/fimmu.2017.01098 28959255 PMC5604070

[10] Harazim M Perrot J Varet H Bourhy H Lannoy J Pikula J. Transcriptomic responses of bat cells to European bat lyssavirus 1 infection under conditions simulating euthermia and hibernation. BMC Immunol (2023) 24:7. doi: 10.1186/s12865-023-00542-7 37085747 PMC10120247

[11] Aicher S-M Streicher F Chazal M Planas D Luo D Buchrieser J. Species-specific molecular barriers to SARS-CoV-2 replication in bat cells. J Virol (2022) 96:e00608–00622. doi: 10.1128/jvi.00608-22 PMC9327701 35862713

[12] Blehert DS Hicks AC Behr M Meteyer CU Berlowski-Zier BM Buckles EL. Bat white-nose syndrome: an emerging fungal pathogen? Science (2009) 323:227–7. doi: 10.1126/science.1163874 18974316

[13] Blehert DS. Fungal disease and the developing story of bat white-nose syndrome. PloS Pathog (2012) 8:e1002779. doi: 10.1371/journal.ppat.1002779 22829763 PMC3400555

[14] Lorch JM Palmer JM Lindner DL Ballmann AE George KG Griffin K. First detection of bat white-nose syndrome in western North America. MSphere (2016) 1:e00148–16. doi: 10.1128/msphere.00148-00116 PMC4973635 27504499

[15] Zukal J Bandouchova H Brichta J Cmokova A Jaron KS Kolarik M. White-nose syndrome without borders: *Pseudogymnoascus destructans* infection tolerated in Europe and Palearctic Asia but not in North America. Sci Rep (2016) 6:19829. doi: 10.1038/srep19829 26821755 PMC4731777

[16] Pikula J Amelon SK Bandouchova H Bartonička T Berkova H Brichta J. White-nose syndrome pathology grading in Nearctic and Palearctic bats. PloS One (2017) 12:e0180435. doi: 10.1371/journal.pone.0180435 28767673 PMC5540284

[17] Pikula J Bandouchova H Novotný L Meteyer CU Zukal J Irwin NR. Histopathology confirms white-nose syndrome in bats in Europe. J Wildl Dis (2012) 48:207–11. doi: 10.7589/0090-3558-48.1.207 22247393

[18] Guivier E Galan M Salvador AR Xuéreb A Chaval Y Olsson GE. Tnf-α expression and promoter sequences reflect the balance of tolerance/resistance to Puumala hantavirus infection in European bank vole populations. Infect Genet Evol (2010) 10:1208–17. doi: 10.1016/j.meegid.2010.07.022 20691810

[19] Kacprzyk J Hughes GM Palsson-Mcdermott EM Quinn SR Puechmaille SJ O'neill LAJ. A potent anti-inflammatory response in bat macrophages may be linked to extended longevity and viral tolerance. Acta Chiropterol (2017) 19:219–28. doi: 10.3161/15081109ACC2017.19.2.001

[20] Meteyer CU Buckles EL Blehert DS Hicks AC Green DE Shearn-Bochsler V. Histopathologic criteria to confirm white-nose syndrome in bats. J Vet Diagn Invest (2009) 21:411–4. doi: 10.1177/104063870902100401 19564488

[21] Bandouchova H Bartonicka T Berkova H Brichta J Cerny J Kovacova V. *Pseudogymnoascus destructans*: evidence of virulent skin invasion for bats under natural conditions, Europe. Transbound Emerg Dis (2015) 62:1–5. doi: 10.1111/tbed.12282 25268034

[22] Cryan PM Meteyer CU Blehert DS Lorch JM Reeder DM Turner GG. Electrolyte depletion in white-nose syndrome bats. J Wildl Dis (2013) 49:398–402. doi: 10.7589/2012-04-121 23568916

[23] Cryan PM Meteyer CU Boyles JG Blehert DS. Wing pathology of white-nose syndrome in bats suggests life-threatening disruption of physiology. BMC Biol (2010) 8:135. doi: 10.1186/1741-7007-8-135 21070683 PMC2984388

[24] Verant ML Meteyer CU Speakman JR Cryan PM Lorch JM Blehert DS. White-nose syndrome initiates a cascade of physiologic disturbances in the hibernating bat host. BMC Physiol (2014) 14:10. doi: 10.1186/s12899-014-0010-4 25487871 PMC4278231

[25] Warnecke L Turner JM Bollinger TK Misra V Cryan PM Blehert DS. Pathophysiology of white-nose syndrome in bats: a mechanistic model linking wing damage to mortality. Biol Lett (2013) 9:20130177. doi: 10.1098/rsbl.2013.0177 23720520 PMC3730627

[26] Warnecke L Turner JM Bollinger TK Lorch JM Misra V Cryan PM. Inoculation of bats with European *Geomyces destructans* supports the novel pathogen hypothesis for the origin of white-nose syndrome. Proc Natl Acad Sci USA (2012) 109:6999–7003. doi: 10.1073/pnas.1200374109 22493237 PMC3344949

[27] O’Donoghue AJ Knudsen GM Beekman C Perry JA Johnson AD Derisi JL. Destructin-1 is a collagen-degrading endopeptidase secreted by *Pseudogymnoascus destructans*, the causative agent of white-nose syndrome. Proc Natl Acad Sci USA (2015) 112:7478–83. doi: 10.1073/pnas.1507082112 PMC4475985 25944934

[28] Veselská T Homutová K García Fraile P Kubátová A Martínková N Pikula J. Comparative eco-physiology revealed extensive enzymatic curtailment, lipases production and strong conidial resilience of the bat pathogenic fungus. Pseudogymnoascus destructans. Sci Rep (2020) 10:16530. doi: 10.1038/s41598-020-73619-7 33020524 PMC7536203

[29] Flieger M Bandouchova H Cerny J Chudíčková M Kolarik M Kovacova V. Vitamin B2 as a virulence factor in *Pseudogymnoascus destructans* skin infection. Sci Rep (2016) 6:33200. doi: 10.1038/srep33200 27620349 PMC5020413

[30] Turner GG Meteyer CU Barton H Gumbs JF Reeder DM Overton B. Nonlethal screening of bat-wing skin with the use of ultraviolet fluorescence to detect lesions indicative of white-nose syndrome. J Wildl Dis (2014) 50:566–73. doi: 10.7589/2014-03-058 24854396

[31] Bandouchova H Bartonička T Berkova H Brichta J Kokurewicz T Kovacova V. Alterations in the health of hibernating bats under pathogen pressure. Sci Rep (2018) 8:6067. doi: 10.1038/s41598-018-24461-5 29666436 PMC5904171

[32] Davy CM Donaldson ME Bandouchova H Breit AM Dorville NA Dzal YA. Transcriptional host–pathogen responses of *Pseudogymnoascus destructans* and three species of bats with white-nose syndrome. Virulence (2020) 11:781–94. doi: 10.1080/21505594.2020.1768018 PMC7549942 32552222

[33] Hecht-Höger AM Braun BC Krause E Meschede A Krahe R Voigt CC. Plasma proteomic profiles differ between European and North American myotid bats colonized by. Pseudogymnoascus destructans. Mol Ecol (2020) 29:1745–55. doi: 10.1111/mec.15437 32279365

[34] Johnson JS Reeder DM Lilley TM Czirják GÁ Voigt CC McMichael JW III. Antibodies to *Pseudogymnoascus destructans* are not sufficient for protection against white-nose syndrome. Ecol Evol (2015) 5:2203–14. doi: 10.1002/ece3.1502 PMC4461422 26078857

[35] Wilkinson GS Brunet-Rossinni A. Methods for age estimation and the study of senescence in bats. In: Ecological and behavioral methods for the study of bats. (Baltimore, Maryland: John Hopkins University Press) (2009). p. 315–25.

[36] Kunz TH Wrazen JA Burnett CD. Changes in body mass and fat reserves in pre-hibernating little brown bats (*Myotis lucifugus*). Ecoscience (1998) 5:8–17. doi: 10.1080/11956860.1998.11682443

[37] Pikula J Heger T Bandouchova H Kovacova V Nemcova M Papezikova I. Phagocyte activity reflects mammalian homeo- and hetero-thermic physiological states. BMC Vet Res (2020) 16:232. doi: 10.1186/s12917-020-02450-z 32631329 PMC7339577

[38] Shuey MM Drees KP Lindner DL Keim P Foster JT. Highly sensitive quantitative PCR for the detection and differentiation of *pseudogymnoascus destructans* and other *pseudogymnoascus* species. Appl Environ Microbiol (2014) 80:1726–31. doi: 10.1128/AEM.02897-13 PMC3957615 24375140

[39] Zukal J Bandouchova H Bartonicka T Berkova H Brack V Brichta J. White-nose syndrome fungus: a generalist pathogen of hibernating bats. PloS One (2014) 9:e97224. doi: 10.1371/journal.pone.0097224 24820101 PMC4018256

[40] Lorch JM Meteyer CU Behr MJ Boyles JG Cryan PM Hicks AC. Experimental infection of bats with *Geomyces destructans* causes white-nose syndrome. Nature (2011) 480:376–8. doi: 10.1038/nature10590 22031324

[41] Puechmaille SJ Wibbelt G Korn V Fuller H Forget F Mühldorfer K. Pan-european distribution of white-nose syndrome fungus (*Geomyces destructans*) not associated with mass mortality. PloS One (2011) 6:e19167. doi: 10.1371/journal.pone.0019167 21556356 PMC3083413

[42] Lockhart SR Guarner J. Emerging and reemerging fungal infections. Semin Diagn Pathol (2019) 36:177–81. doi: 10.1053/j.semdp.2019.04.010 PMC11979780 31010605

[43] Zukal J Berková H Banďouchová H Kováčová V Pikula J. Bats and caves: activity and ecology of bats wintering in caves. In: Cave investigation. Rijeka: InTech (2017). p. 51–75.

[44] Haase CG Fuller NW Dzal YA Hranac CR Hayman DTS Lausen CL. Body mass and hibernation microclimate may predict bat susceptibility to white-nose syndrome. Ecol Evol (2021) 11:506–15. doi: 10.1002/ece3.7070 PMC7790633 33437446

[45] Tada T Okumura K Takemori T Arai T. Immunogenicity of fungi for the production of reaginic antibody in the rat. Allerg Immunol (Leipz) (1974) 20-21:427–34. 4377863

[46] Brennan RE Caire W Pugh N Chapman S Robbins AH Akiyoshi DE. Examination of bats in western Oklahoma for antibodies against *Pseudogymnoascus destructans*, the causative agent of White-Nose Syndrome. Southwestern Nat (2015) 60:145–150, 146. doi: 10.1894/SWNAT-D-14-00030.1

[47] Carvalho A Cunha C Iannitti R Casagrande A Bistoni F Aversa F. Host defense pathways against fungi: the basis for vaccines and immunotherapy. Front Microbiol (2012) 3:176. doi: 10.3389/fmicb.2012.00176 22590466 PMC3349272

[48] Rocke TE Kingstad-Bakke B Wüthrich M Stading B Abbott RC Isidoro-Ayza M. Virally-vectored vaccine candidates against white-nose syndrome induce anti-fungal immune response in little brown bats (*Myotis lucifugus*). Sci Rep (2019) 9:6788. doi: 10.1038/s41598-019-43210-w 31043669 PMC6494898

[49] Willis CKR. Trade-offs influencing the physiological ecology of hibernation in temperate-zone bats. Integr Comp Biol (2017) 57:1214–24. doi: 10.1093/icb/icx087 28985332

[50] Voigt CC Fritze M Lindecke O Costantini D Pētersons G Czirják GÁ. The immune response of bats differs between pre-migration and migration seasons. Sci Rep (2020) 10:17384. doi: 10.1038/s41598-020-74473-3 33060711 PMC7562910

[51] Carey HV Andrews MT Martin SL. Mammalian hibernation: cellular and molecular responses to depressed metabolism and low temperature. Physiol Rev (2003) 83:1153–81. doi: 10.1152/physrev.00008.2003 14506303

[52] Xu Y Shao C Fedorov VB Goropashnaya AV Barnes BM Yan J. Molecular signatures of mammalian hibernation: comparisons with alternative phenotypes. BMC Genomics (2013) 14:567. doi: 10.1186/1471-2164-14-567 23957789 PMC3751779

[53] Boyer BB Barnes BM. Molecular and Metabolic Aspects of Mammalian Hibernation: Expression of the hibernation phenotype results from the coordinated regulation of multiple physiological and molecular events during preparation for and entry into torpor. BioScience (1999) 49:713–24. doi: 10.2307/1313595

[54] Webb PI Speakman JR Racey PA. How hot is a hibernaculum? A review of the temperatures at which bats hibernate. Can J Zool (1996) 74:761–5. doi: 10.1139/z96-087

[55] Perry RW. A review of factors affecting cave climates for hibernating bats in temperate North America. Environ Rev (2013) 21:28–39. doi: 10.1139/er-2012-0042

[56] Meteyer CU Barber D Mandl JN. Pathology in euthermic bats with white nose syndrome suggests a natural manifestation of immune reconstitution inflammatory syndrome. Virulence (2012) 3:583–8. doi: 10.4161/viru.22330 PMC3545935 23154286

[57] Bouma HR Carey HV Kroese FGM. Hibernation: the immune system at rest? J Leukoc Biol (2010) 88:619–24. doi: 10.1189/jlb.0310174 20519639

[58] Bouma HR Dugbartey GJ Boerema AS Talaei F Herwig A Goris M. Reduction of body temperature governs neutrophil retention in hibernating and nonhibernating animals by margination. J Leukoc Biol (2013) 94:431–7. doi: 10.1189/jlb.0611298 23766528

[59] Bouma HR Kroese FG Kok JW Talaei F Boerema AS Herwig A. Low body temperature governs the decline of circulating lymphocytes during hibernation through sphingosine-1-phosphate. P Natl Acad Sci USA (2011) 108:2052–7. doi: 10.1073/pnas.1008823108 PMC3033260 21245336

[60] Heger T Zukal J Seidlová V Němcová M Necas D Papežíková I. Measurement of phagocyte activity in heterotherms. Acta Vet Brno (2020) 89:79–87. doi: 10.2754/avb202089010079

[61] Nemcova M Seidlova V Zukal J Dundarova H Zukalova K Pikula J. Performance of bat-derived macrophages at different temperatures. Front Vet Sci (2022) 9:978756. doi: 10.3389/fvets.2022.978756 36157196 PMC9500541

[62] Bouma HR Strijkstra AM Talaei F Henning RH Carey HV Kroese FGM. The hibernating immune systém. In: Ruf T Bieber C Arnold W Millesi E, editors. Living in a seasonal world: thermoregulatory and metabolic adaptations. Berlin, Heidelberg: Springer Berlin Heidelberg (2012). p. 259–70.

[63] Wüthrich M Deepe GS Klein B. Adaptive immunity to fungi. Annu Rev Immunol (2012) 30:115–48. doi: 10.1146/annurev-immunol-020711-074958 PMC3584681 22224780

[64] Blanco JL Garcia ME. Immune response to fungal infections. Vet Immunol Immunopathol (2008) 125:47–70. doi: 10.1016/j.vetimm.2008.04.020 18565595

[65] Casadevall A Pirofski L-A. Immunoglobulins in defense, pathogenesis, and therapy of fungal diseases. Cell Host Microbe (2012) 11:447–56. doi: 10.1016/j.chom.2012.04.004 PMC3360875 22607798

[66] Rosales C Demaurex N Lowell CA Uribe-Querol E. Neutrophils: their role in innate and adaptive immunity. J Immunol Res (2016) 2016:1469780. doi: 10.1155/2016/1469780 27006954 PMC4783580

[67] Field KA Johnson JS Lilley TM Reeder SM Rogers EJ Behr MJ. The white-nose syndrome transcriptome: activation of anti-fungal host responses in wing tissue of hibernating little brown myotis. PloS Pathog (2015) 11:e1005168. doi: 10.1371/journal.ppat.1005168 26426272 PMC4591128

[68] Donaldson ME Davy CM Willis CKR Mcburney S Park A Kyle CJ. Profiling the immunome of little brown myotis provides a yardstick for measuring the genetic response to white-nose syndrome. Evol Appl (2017) 10:1076–90. doi: 10.1111/eva.12514 PMC5680615 29151862

[69] Fritze M Costantini D Fickel J Wehner D Czirják GÁ Voigt CC. Immune response of hibernating European bats to a fungal challenge. Biol Open (2019) 8:bio046078. doi: 10.1242/bio.046078 31649120 PMC6826279

[70] Reeder SM Palmer JM Prokkola JM Lilley TM Reeder DM Field KA. *Pseudogymnoascus destructans* transcriptome changes during white-nose syndrome infections. Virulence (2017) 8:1695–707. doi: 10.1080/21505594.2017.1342910 PMC5810475 28614673

[71] Santamaría R Rizzetto L Bromley M Zelante T Lee W Cavalieri D. Systems biology of infectious diseases: a focus on fungal infections. Immunobiology (2011) 216:1212–27. doi: 10.1016/j.imbio.2011.08.004 21889228

[72] O’Connor CM Cooke SJ. Ecological carryover effects complicate conservation. Ambio (2015) 44:582–91. doi: 10.1007/s13280-015-0630-3 PMC4552714 25678024

[73] Harrison XA Blount JD Inger R Norris DR Bearhop S. Carry-over effects as drivers of fitness differences in animals. J Anim Ecol (2011) 80:4–18. doi: 10.1111/j.1365-2656.2010.01740.x 20726924

[74] Jonasson KA Willis CKR. Changes in body condition of hibernating bats support the thrifty female hypothesis and predict consequences for populations with white-nose syndrome. PloS One (2011) 6:e21061. doi: 10.1371/journal.pone.0021061 21731647 PMC3120823

[75] Speakman JR. The physiological costs of reproduction in small mammals. Philos Trans R Soc B: Biol Sci (2008) 363:375–98. doi: 10.1098/rstb.2007.2145 PMC2606756 17686735

[76] Francl KE Ford WM Sparks DW Brack V. Capture and reproductive trends in summer bat communities in west virginia: assessing the impact of white-nose syndrome. J Fish Wildl Manag (2012) 3:33–42. doi: 10.3996/062011-jfwm-039

[77] Davy CM MastroMonaco GF Riley JL Baxter-Gilbert JH Mayberry H Willis CKR. Conservation implications of physiological carry-over effects in bats recovering from white-nose syndrome. Conserv Biol (2017) 31:615–24. doi: 10.1111/cobi.12841 27641049

[78] Martínková N Pikula J Zukal J Kovacova V Bandouchova H Bartonička T. Hibernation temperature-dependent *Pseudogymnoascus destructans* infection intensity in Palearctic bats. Virulence (2018) 9:1734–50. doi: 10.1080/21505594.2018.1548685 PMC10022473 36595968

